# Three-Dimensional Culture Model of Skeletal Muscle Tissue with Atrophy Induced by Dexamethasone

**DOI:** 10.3390/bioengineering4020056

**Published:** 2017-06-15

**Authors:** Kazunori Shimizu, Riho Genma, Yuuki Gotou, Sumire Nagasaka, Hiroyuki Honda

**Affiliations:** 1 Department of Biomolecular Engineering, Graduate School of Engineering, Nagoya University, Nagoya 464-8603, Japan; genma.riho@k.mbox.nagoya-u.ac.jp (R.G.); gotou.yuuki@a.mbox.nagoya-u.ac.jp (Y.G.); nagasaka.sumire@f.mbox.nagoya-u.ac.jp (S.N.); honda@chembio.nagoya-u.ac.jp (H.H.); 2 Innovative Research Center for Preventive Medical Engineering, Nagoya University, Nagoya 464-8601, Japan

**Keywords:** cell-based assay, disease model, drug screening, contractile force, microdevice

## Abstract

Drug screening systems for muscle atrophy based on the contractile force of cultured skeletal muscle tissues are required for the development of preventive or therapeutic drugs for atrophy. This study aims to develop a muscle atrophy model by inducing atrophy in normal muscle tissues constructed on microdevices capable of measuring the contractile force and to verify if this model is suitable for drug screening using the contractile force as an index. Tissue engineered skeletal muscles containing striated myotubes were prepared on the microdevices for the study. The addition of 100 µM dexamethasone (Dex), which is used as a muscle atrophy inducer, for 24 h reduced the contractile force significantly. An increase in the expression of *Atrogin-1* and *MuRF-1* in the tissues treated with Dex was established. A decrease in the number of striated myotubes was also observed in the tissues treated with Dex. Treatment with 8 ng/mL Insulin-like Growth Factor (IGF-I) for 24 h significantly increased the contractile force of the Dex-induced atrophic tissues. The same treatment, though, had no impact on the force of the normal tissues. Thus, it is envisaged that the atrophic skeletal muscle tissues induced by Dex can be used for drug screening against atrophy.

## 1. Introduction

Skeletal muscle atrophy and muscle mass loss weaken muscular strength, resulting in impairment of capacity for physical exercise. Skeletal muscle atrophy includes disuse muscle atrophy caused by a lack of physical exercise, being bedridden, sarcopenia due to aging, cachexia caused by cancer, and steroid myopathy due to excessive glucocorticoid presence [1,2,3]. Skeletal muscles account for approximately half of the total body weight of adults and are the organs involved in motion and posture maintenance. Moreover, they play a crucial role in energy production and carbohydrate metabolism. Therefore, the functional deterioration of skeletal muscles directly causes a reduction in exercise capacity as well as a metabolic abnormality, in turn deteriorating the quality of life significantly. Currently, because of the non-availability of a fundamental treatment for muscle atrophy, prevention and treatment by counter-measure therapies involving proper diet and exercise are performed [4,5]. However, the development of direct preventive/therapeutic drugs to counter muscle atrophy is urgently required.

Cell assays using skeletal muscle cells cultured in two-dimensional (2D) plane culture are used for drug development [6,7,8,9]. The drug candidate compound is added to these skeletal muscle cells, and the increase or decrease in the gene/protein expression of molecules related to muscle differentiation, such as the myosin heavy chain, is used as a screening index. In addition, an alteration in the size of a muscle fiber due to the addition of candidate compounds is also used as a screening index. However, variations in the expression levels of genes/proteins as well as the size of muscle fibers are insufficient for appropriate indication of muscle contractile force. This is because the skeletal muscle contractile force is exerted due to the complex interaction of multiple second-messenger pathways and structural proteins.

In recent years, with the progress of tissue engineering, microfluidic systems, and micro-electromechanical systems (MEMS), a number of techniques have been developed for constructing contractile skeletal muscle cells/tissues and for quantifying their contractions [10,11,12,13,14,15,16,17,18,19,20,21]. These techniques have also been applied to drug screening using the contractile force as an indicator. For instance, Vandenburgh et al. developed a microdevice incorporating two silicone rubber posts, constructed a three-dimensional (3D) muscle tissue on the device, and developed a 96-well-format drug screening system using muscle contractile force as an index [13]. Furthermore, they constructed muscle tissue on the device from myoblasts of the mdx murine model of Duchenne muscular dystrophy (DMD) and carried out drug screening [22]. Thus, drug screening using muscle contractile force as an indicator is considered to be effective. Nevertheless, the number of drug screening systems developed based on contractile force for muscle atrophy is low. It is reckoned that a versatile and convenient drug screening system for muscle atrophy would be realized if atrophic muscle tissue can be constructed using normal skeletal muscle cells by regulating culture conditions.

In the present study, we aimed to develop a muscle atrophy model by inducing atrophy in a normal muscle tissue constructed on the microdevice. The model should be capable of measuring contractile force and to verify if it is suited to drug screening applications, where contractile force is used as an index. Glucocorticoids trigger the skeletal muscle atrophy observed in many pathological states such as sepsis, cachexia, starvation, metabolic acidosis, and severe insulinopenia [2]. Dexamethasone (Dex), a type of glucocorticoid drug, is known to induce steroid myopathy due to the formation of excessive glucocorticoids as a side effect of the treatment [2]. In the present study, Dex was used as a muscle atrophy inducer. It has been used for inducing muscle atrophy in 2D cultured skeletal muscle cells in vitro [8,23,24]. Dex treatment induced the expression of skeletal muscle atrophy-related genes, *Atrogin-1* and *MuRF-1*, in myotubes and decreased the size of myotubes and the amount of muscle protein. In this study, 3D skeletal muscle tissues were constructed on the microdevice capable of measuring their contractile force and treated with Dex. For the first time, it was determined that their contractile force decreased with the treatment, which established that Dex-induced atrophic 3D muscle tissue can potentially be used for drug screening.

## 2. Materials and Methods

### 2.1. Microdevice Design and Fabrication

**Figure 1:**
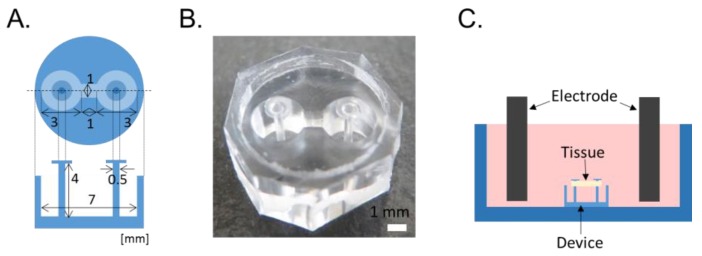
(**A**) Configuration of the microdevice, (**B**) Image of the fabricated device, (**C**) Schematic diagram of skeletal muscle tissue culture and electrical stimulation.

The microdevice used for constructing the skeletal muscle tissues and quantifying their contractile force was developed as previously reported with certain modifications [13]. In the previous study, the device with a circular pocket was used. Meanwhile, in the present study, we modified the shape of the pocket of the device from the circle to the dumbbell because the tearing/breaking of the constructed tissues was rarely observed when we used the device with the dumbbell-shaped pocket. The size and design of the device are illustrated in Figure 1A. The device has a dumbbell-shaped pocket with a depth of 3 mm and the pocket has two flexible micro-posts, each of diameter 500 µm. The device was fabricated by molding polydimethylsiloxane (PDMS; SILPOT 184, Dow Corning Toray, Tokyo, Japan) using a Teflon mold. The Teflon mold was immersed in a 10:1 mixture of pre-cured PDMS, placed in a vacuum chamber to remove air bubbles, and baked at 70 °C for 60 min. Subsequently, the mold was removed and the cured PDMS was cut into individual devices. A circular PDMS thin membrane 1.5 mm in diameter was attached to the tip of the micro-post using pre-cured PDMS as an adherent, and the pre-cured PDMS was cured at 70 °C for 60 min (Figure 1B).

### 2.2. Cell Culture

C2C12—mouse skeletal muscle myoblasts, were cultured in Dulbecco’s Modified Eagle Medium (DMEM) (08458-16, Nacalai Tesque, Kyoto, Japan) supplemented with 10% fetal bovine serum (Invitrogen, Gaithersburg, MD, USA) and 1% Penicillin-Streptomycin (PS, Invitrogen) (growth medium; GM). The cells were maintained at 37 °C under 5% CO_2_, and the medium was replaced each day with a fresh medium. The passage was conducted when the cells reached 80–90% confluency.

### 2.3. Construction of Skeletal Muscle Tissues

The microdevices were installed at the center of a well of a 6-well plate using silicone adhesive seals (NSC-100, Nippa, Osaka, Japan) and sterilized under UV for 1 h. The dumbbell shaped pocket on the device was filled with 2% Pluronic F-127 solution (P6867, Invitrogen) at 4 °C to inhibit the attachment of the tissue to the surface. Twenty-four hours later, the solution was aspirated, and the device was used for constructing skeletal muscle tissues.

The muscle tissues were constructed as reported previously [25]. The total volume of the cell-hydrogel mixture was defined as *V*. Ice-cold fibrinogen from bovine plasma (10 mg/mL, F8630, Sigma-Aldrich, Darmstadt, Germany), Matrigel (354234, Corning, New York, NY, USA), and 2 × DMEM were mixed in a micro-tube on ice with a volume ratio of 0.2:0.1:0.2. Then, 0.484 *V* of GM containing myoblasts of concentration 2 × 10^6^ cells/mL was added to 0.5 *V* of the hydrogel mixture. Finally, 0.016 *V* of thrombin from bovine plasma (50 U/mL, T4648, Sigma-Aldrich) was added to the mixture. 45 µL of the resulting cell and hydrogel solution was poured into the dumbbell shaped pocket on the device and incubated at 37 °C to solidify the hydrogel.

The tissue was cultured in GM for two days. Subsequently, the medium was altered to a differentiation medium (DM) DMEM containing 2% horse serum and 1% Penicillin-Streptomycin, for six days. All the culture media contained 2.0 mg/mL 6-Aminocaproic acid (A2504, Sigma-Aldrich) to prevent disassembly of the tissues. The medium was replaced every three days.

### 2.4. Contractile Force Measurement of the Muscle Tissues

The muscle tissues constructed on the device in a well of a 6-well plate were then electrically stimulated to achieve maximum tetanic force (Figure 1C). An electrical stimulus of 20 V at 30 Hz with 2 ms wide pulses (C-Pace EP, IonOptix, Westwood, MA, USA) and electrodes for the 6-well plate (C-CLD6WACN, IonOptix) were used. The displacement of the tip of the micro-posts was observed by using an upright microscope with a 20 times magnification (BX53F, Olympus, Osaka, Japan). The electrical stimulation was applied to each tissue for 5 s, and the images were captured both without and with the stimulation, and the displacement was measured.

The contractile force was calculated using the formulas described previously [13].

I = (1/4)πR^4^, (1)

F = 3EIδ/L^3^ = 3πER^4^δ/(4L^3^), (2)

where I = moment of inertia, R = radius of a micro-post (0.5 mm), L = length of a micro-post (4 mm), E = elastic modulus of PDMS (1.7 MN/m^2^), and δ = displacement of the tip of a micro-post.

### 2.5. Treatment with Dexamethasone and IGF-I

Dexamethasone (Dex, Sigma-Aldrich) was used to induce atrophy in the constructed skeletal muscle tissue on the device. Dex was diluted in ethanol to a concentration of 20 mmol/mL and was mixed with the DM to obtain final concentrations of 50, 100, and 200 µM [26]. The final ethanol concentration was 1% or lower. On day 6, muscle tissues on the device were treated with Dex by medium alteration and cultured for the subsequent experiments.

To verify the contractile force recovery effect, a recombinant human insulin-like growth factor -I (IGF-I) (AF-100-11, Peprotech, Rocky Hill, NJ, USA), was used. IGF-I was diluted in DM and used at a final concentration of 8 ng/mL.

### 2.6. Real-Time PCR

The total RNA was extracted from the muscle tissues using Nucleospin RNA (740955.50, MACHEREY-NAGEL, Duren, Germany) by following the manufacturer’s instructions. First-strand cDNA was prepared from the extracted RNA by using ReverTra Ace qPCR RT Master Mix with gDNA Remover (FSQ-301, Toyobo, Osaka, Japan), according to the manufacturer’s instructions. Real-Time PCR was performed by Eco Real-Time PCR system (Illumina, San Diego, CA, USA) using THUNDERBIRD SYBR qPCR Mix (QPS-201, Toyobo). The conditions for real-time PCR were as follows: (1) 95 °C for 1 min, (2) 95 °C for 15 s, (3) 60 °C for 30 s, and (4) 45 cycles of (2) and (3). The melting curve was measured from 55 °C to 95 °C. Primers for PCR were purchased from FASMAC (Kanagawa, Japan). The sequences of these primers are as follows: mouse *Atrogin-1* forward: 5′-GGCGGACGGCTGGAA-3′, mouse *Atrogin-1* reverse: 5′-CAGATTCTCCTTACTGTATACCTCCTTGT-3′, mouse *MuRF-1* forward: 5′-ACGAGAAGAAGAGCGAGCTG-3′, *MuRF-1* reverse: 5′-CTTGGCACTTGAGAGAGGAAGG-3′, mouse *β-actin* forward: 5′-CGTTGACATCCGTAAAGACCTC-3′, and mouse *β-actin* reverse: 5′-AGCCACCGATCCACACAGA-3′.

### 2.7. Fluorescent Immunostaining

The tissue was left in 4% para-formaldehyde (163-20145, Wako, Osaka, Japan) for 2 h, washed three times for 10 min each with PBS, and permeabilized for 10 min with 0.3% Triton X-100/PBS (T8787, Sigma-Aldrich). Subsequently, the tissue was washed three times for 10 min each with PBS and blocked with blocking solution (10% GS, 0.01% TritonX-100/PBS) for 30 min at room temperature. The tissue was incubated with the primary antibody (anti-fast MHC, ab91506, Abcam) for 3 h at room temperature and then washed three times for 10 min each with PBS. Then, it was incubated with the secondary antibody, CF488A Goat Anti-Rabbit IgG (H + L) (20012, Biotium, Fremont, CA, USA), and 4′,6-diamidino-2-phenylindole (DAPI) for 2 h at room temperature and washed three times for 10 min each with PBS. The tissue was observed using BZ-X700 (KEYENCE, Osaka, Japan).

The fluorescent images were used for measuring the population of myotubes with the sarcomere structure, as reported previously [27]. Tubular cells with more than five nuclei were counted as myotubes and cells with striations were scored as positive.

### 2.8. Statistical Analysis

Statistical analysis was performed using a t-test for independent samples with unequal variance using the Prism 5 software (Graphpad Software, LaJolla, CA, USA).

## 3. Results and Discussion

### 3.1. Construction of Skeletal Muscle Tissues on Devices

**Figure 2:**
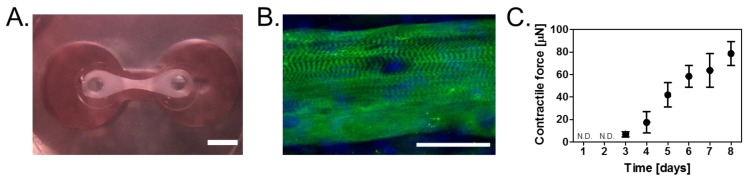
(**A**) Top view image of a skeletal muscle tissue constructed on the device. The scale bar reads 1 mm; (**B**) Fluorescence micrograph of a myotube in the skeletal muscle tissue on the device, which was cultured in DM for six days. Here, green indicates fast MHC and blue indicates the nuclei. The scale bar reads 50 µm; (**C**) Time course of contractile force after culturing in DM. Results represent mean ± SD (*n* = 3). N.D. indicates not detected.

Skeletal muscle tissue was prepared on the microdevices for measuring muscle contractile forces. The tissue shifted to the upper parts of the posts on the first day of growth culture and formed a ribbon-shaped tissue by self-organization (Figure 2A). The tissue contained several myotubes with sarcomere structure, which were aligned along the long axis direction of the tissue (Figure 2B).

After differentiation was induced (day 0), the contractile force of the muscle tissues with respect to the number of days elapsed was measured (Figure 2C). A contractile force of 6.6 ± 2.2 μN was observed from the third day of differentiation, which increased to 77.5 ± 11.1 μN by the eighth day of differentiation (Figure 2C). As a sufficiently stable contractile force was obtained on the sixth day (57.5 ± 12.8 μN) as reported previously [13], it was decided that muscle tissue prepared by differentiation culture for six days is to be used in subsequent experiments.

### 3.2. Effect of Dex on Contractile Force of the Skeletal Muscle Tissues

**Figure 3:**
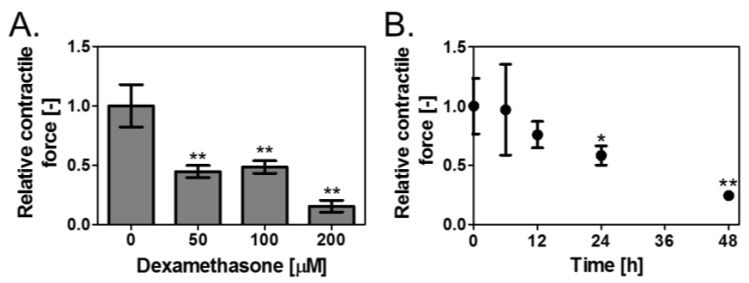
Loss of contractile force of skeletal muscle tissues by treatment with Dex. (**A**) Effects of Dex concentration (0, 50, 100, and 200 µM) on contractile force of the skeletal muscle tissues cultured in DM for 6 d. The tissues were treated with Dex for 48 h. Results represent mean ± SD (*n* = 3). ** *p* < 0.01 versus 0 µM; (**B**) Effects of Dex treatment duration (0, 6, 12, 24, and 48 h) on contractile force of the skeletal muscle tissues cultured in DM for 6 d. Dex was added at 100 µM. Results represent mean ± SD (*n* = 3). * *p* < 0.05 versus 0 h. ** *p* < 0.01 versus 0 h.

Next, investigations were carried out to determine if the muscle contractile force of the muscle tissue produced is decreased by Dex. Dex was added at 0, 50, 100, and 200 μM to the muscle tissue on the device, prepared by a differentiation culture lasting six days, and the alteration in the contractile force was measured after 48 h. This contractile force was compared to that of the muscle tissue to which Dex was not added. As a result, it was revealed that the contractile force significantly decreased in the muscle tissue to which Dex was added (0.45 ± 0.05 for 50 μM, 0.48 ± 0.05 for 100 μM, 0.15 ± 0.05 for 200 μM) (Figure 3A). Furthermore, the variation of the contractile force with time after the addition of 100 μM Dex was investigated. The contractile force decreased significantly after 24 h (0.58 ± 0.08) and reached 0.24 ± 0.03 after 48 h (Figure 3B). Thus, it was determined that the contractile force of the cultured three-dimensional skeletal muscle tissue was significantly decreased by adding Dex.

### 3.3. Evaluation of Muscle Tissue with Reduced Contractile Force Due to Addition of Dex

**Figure 4:**
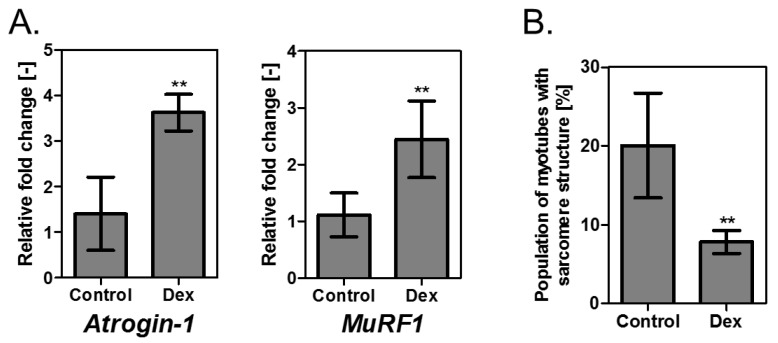
Characterization of atrophic muscle tissues induced by Dex. (**A**) *Atrogin-1* and *MuRF-1* gene expression levels in muscle tissues treated with 100 µM Dex for 24–48 h are expressed relative to *β-actin*. mRNA was extracted from nine tissues each for the control and Dex. Results represent mean ± SD (*n* = 5) **p < 0.01 versus control; (**B**) Percentage of myotubes with the sarcomere structure in muscle tissues treated with 100 µM Dex for 24 h. Results represent mean ± SD (*n* = 6). Totally 190 myotubes were analyzed for control, and 136 myotubes were analyzed for Dex. **p < 0.01 versus control.

The addition of Dex to skeletal muscle cells cultured in 2D is known to increase the expression of atrogenes including ubiquitin ligases *Atrogin-1* and muscle-specific RING-Finger protein, *MuRF-1* [24]. The expression profile of these genes in the skeletal muscle tissues with reduced contractile force (treated with 100 μM Dex for 24–48 h) was examined. The obtained values were normalized using *β-actin*. Compared with the control, expression levels increased significantly by 2.6 times for *Atrogin-1* and 2.2 times for *MuRF-1* because of Dex addition (Figure 4A).

As the increased expression of *Atrogin-1* and *MuRF-1* by Dex addition was established, it was presumed that muscle protein degradation was promoted in these muscle tissues.

Next, it was examined if the number of myotubes retaining the sarcomere structure is altered by the addition of Dex. As a result, it was determined that compared to the control state (without the addition of Dex), the proportion of myotubes retaining the sarcomere structure was significantly lower in the muscle tissue 24 h after the addition of Dex (Figure 4B). It has been reported that periodic electrical pulse stimulation of myotubes significantly increases the proportion of myotubes retaining the sarcomere structure [27,28,29,30]. Furthermore, it is reported that interruption of electrical stimulation causes the sarcomere structure to disappear in a short time and the contractile force of the myotubes to decreases sharply [27]. Although it is not evident how the addition of Dex decreases the proportion of sarcomeric myotubes in a three-dimensional muscle tissue (Figure 4B), this phenomenon is believed to be one of the factors affecting the decrease in muscle contractile force upon the addition of Dex.

Thus, these results suggested that proteolytic degradation of the ubiquitin-proteasome system and alterations in sarcomere structure is likely to be involved in the reduction of contractile force due to Dex addition and that Dex effectively induces atrophy in 3D skeletal muscle tissue.

### 3.4. Verification of Contractile Force Recovery Effect by Addition of IGF-I to Muscle Atrophy Model

Finally, in order to investigate if the atrophic muscle tissues induced by Dex can be applied to drug screening, it was verified if the contractility was recovered by adding the model compound IGF-I. IGF-I is a peptide growth hormone and plays a major role in promoting skeletal muscle growth and differentiation [31]. It has been reported elsewhere that IGF-I increases contractile force of engineered normal skeletal muscle tissues [13,32,33]. It has also been reported that IGF-I prevents a Dex-induced increase in proteolysis and blocks Dex induction of atrogenes, including *Atrogin-1* and *MuRF-1,* in C2C12 myotubes [34]. Thus, although it is not evident that IGF-I prevents Dex-induced decrease of contractile force of atrophic tissues, it was used as a model compound in this study.

**Figure 5:**
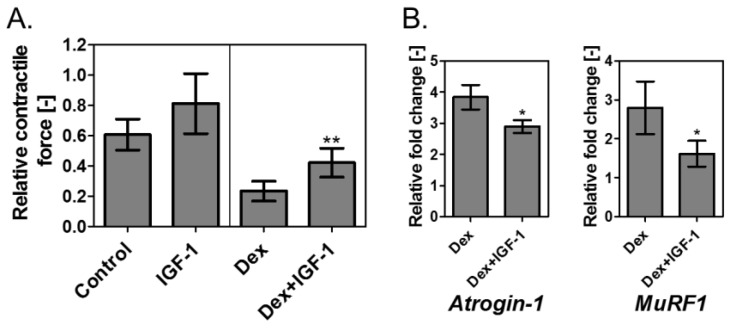
Effects of IGF-I addition on atrophic skeletal muscles. (**A**) Variation in contractile force of atrophic tissues by the addition of Dex. The muscle tissues on the device prepared by six-day differentiation culture (0 h) were treated with 100 µM Dex for 48 h (Dex group). For Dex + IGF-I group, 8 ng/mL IGF-I was added at 24 h and incubated for the subsequent 24 h. Muscle contractile force was measured at 48 h. The relative value was determined with the contractile force at 0 h designated as 1. For the control and IGF-I groups, similar volume of ethanol was used instead of Dex. Results represent mean ± SD (*n* = 6). ** *p* < 0.01 versus Dex; (**B**) *Atrogin-1* and *MuRF-1* gene expression levels in muscle tissues treated with 100 µM Dex for 24 h, and 100 µM Dex and 8 ng/mL IGF-I for subsequent 24 h are expressed relative to *β-actin*. Results represent mean ± SD (*n* = 3). * *p* < 0.05 versus Dex.

100 μM Dex was added to the muscle tissue on the device, prepared by a differentiation culture for 24 h, to induce atrophy. Then, 8 ng/mL IGF-I was added. The mixture was incubated for 24 h, and muscle contractile force was measured (Figure 5A). The relative value was determined by assigning the value 1 to the contractile force before addition of Dex. As a result, the group to which only Dex was added yielded a relative value of 0.23 ± 0.07, whereas the group to which both Dex and IGF-I were added yielded 0.42 ± 0.10. Thus, any decrease in contractile force was not completely eliminated but significantly reduced by the addition of IGF-I to the Dex-treated tissue (*p* < 0.01) (Figure 5A). It should be elucidated in the future whether the treatment with IGF-I for longer than 24 h fully recovers the contractile force of the Dex-treated tissue.

In addition, variations in the expression levels of atrogenes in Dex and Dex + IGF-I were examined (Figure 5B). The expression level of each gene in Dex + IGF-I was significantly lower compared to that in Dex (*Atrogin-1*: 3.83 ± 0.39 for Dex and 2.89 ± 0.20 for Dex + IGF-I; *MuRF-1*: 2.79 ± 0.68 for Dex and 1.61 ± 0.33 for Dex + IGF-I). These results indicate that IGF-I suppresses muscle weakness by reducing the proteolytic effect of ubiquitin proteasome, induced by Dex addition through the phosphatidylinositol 3-kinase (PI3K)-Akt pathway [35]. Thus, the atrophic muscle tissues induced by Dex are suitable for the application of screening the drugs.

As illustrated in Figure 5A, when IGF-I was added to the normal skeletal muscle tissues, the contractile force displayed a tendency to increase, albeit not significantly (0.61 ± 0.10 for control and 0.81 ± 0.20 for IGF-I). On the contrary, in previous studies, it was reported that IGF-I increased the contractile force of engineered normal skeletal muscle tissue; Vandenburgh et al. reported that the contractile force of the fibrin gel-based skeletal muscle tissue was significantly increased two days after the addition of IGF-I to the medium at a concentration of 100 ng/mL [13]; Huang et al. directly embedded IGF-I in the fibrin gel (25 ng/mL) and reported that it results in a significant increase in contractile force—50% over untreated tissues—after 14 days [33]; and Sato et al. constructed skeletal muscle tissue using IGF-I gene-engineered myoblast cells, which secreted IGF-I at approximately 6.5 ng/(mL·d) to the medium and reported that the tissue on day 7 generated significantly higher (1.5 times) contractile force than the control [32]. In the present study, IGF-I was added to the tissue at 8 ng/mL for 24 h, and no significant increase in the contractile force was observed (Figure 5A). Comparing these results, it can be concluded that addition of IGF-I is likely to be marginally effective in increasing the contractile force of normal skeletal muscle tissues under shorter culture durations. Therefore, although further investigation is necessary to examine the effects of longer culture durations on the contractile force, by using skeletal muscle tissues, in which atrophy was induced by adding Dex, high sensitivity screening is likely to be possible in a shorter period than by using normal skeletal muscle tissue.

In the present study, we suggest the usability of the Dex-induced atrophic tissues for drug screening by using mice C2C12 cells (Figure 5). In the future, it is expected that human skeletal muscle cells, including primary cells and the cells derived from induced pluripotent stem cells (iPSCs) [36,37,38], will be used. It has been reported that Dex induced the expression of *Atrogin-1* and *MuRF-1* in primary human myotubes [39]. Thus, the system used in this study would be applicable to the human cells although the optimization of the process for constructing tissues and inducing atrophy should be examined. Furthermore, miniaturization of the system, especially the devices and electrodes, will be needed to improve the throughput. Nonetheless, since the device fabrication, tissue construction and atrophy induction were simple, and the equipment for the electrical stimulation was commercially available, we believe that the system used in this study shows high versatility.

## 4. Conclusions

This study reports the development of a muscle atrophy model by inducing atrophy in normal muscle tissue constructed on a microdevice capable of measuring contractile force. The addition of Dex, used as an atrophy inducer in this study, to the tissues increases the expression of atrogenes. It also decreases the contractile force of the tissues. A suppression of loss of contractile force was observed following the treatment of the Dex-induced atrophic muscle tissues with IGF-I. Thus, the atrophic muscle tissues are believed to be useful for drug screening against muscle atrophy.

## References

[1] Bonaldo P., Sandri M. (2013). Cellular and molecular mechanisms of muscle atrophy. Dis. Model. Mech..

[2] Schakman O., Kalista S., Barbe C., Loumaye A., Thissen J.P. (2013). Glucocorticoid-induced skeletal muscle atrophy. Int. J. Biochem. Cell Biol..

[3] Thomas D.R. (2007). Loss of skeletal muscle mass in aging: Examining the relationship of starvation, sarcopenia and cachexia. Clin. Nutr..

[4] Murphy C.H., Churchward-Venne T.A., Mitchell C.J., Kolar N.M., Kassis A., Karagounis L.G., Burke L.M., Hawley J.A., Phillips S.M. (2015). Hypoenergetic diet-induced reductions in myofibrillar protein synthesis are restored with resistance training and balanced daily protein ingestion in older men. Am. J. Physiol. Endocrinol. Metab..

[5] Lewelt A., Krosschell K.J., Stoddard G.J., Weng C.D., Xue M., Marcus R.L., Gappmaier E., Viollet L., Johnson B.A., White A.T. (2015). Resistance strength training exercise in children with spinal muscular atrophy. Muscle Nerve.

[6] Hemdan D.I., Hirasaka K., Nakao R., Kohno S., Kagawa S., Abe T., Harada-Sukeno A., Okumura Y., Nakaya Y., Terao J. (2009). Polyphenols prevent clinorotation-induced expression of atrogenes in mouse C2C12 skeletal myotubes. J. Med. Investig..

[7] Kawai N., Hirasaka K., Maeda T., Haruna M., Shiota C., Ochi A., Abe T., Kohno S., Ohno A., Teshima-Kondo S. (2015). Prevention of skeletal muscle atrophy in vitro using anti-ubiquitination oligopeptide carried by atelocollagen. Biochim. Biophys. Acta (BBA) Mol. Cell. Res..

[8] Alamdari N., Aversa Z., Castillero E., Gurav A., Petkova V., Tizio S., Hasselgren P.O. (2012). Resveratrol prevents dexamethasone-induced expression of the muscle atrophy-related ubiquitin ligases atrogin-1 and murf1 in cultured myotubes through a sirt1-dependent mechanism. Biochem. Biophys. Res. Commun..

[9] Wang D.T., Yin Y., Yang Y.J., Lv P.J., Shi Y., Lu L., Wei L.B. (2014). Resveratrol prevents tnf-alpha-induced muscle atrophy via regulation of akt/mtor/foxo1 signaling in C2C12 myotubes. Int. Immunopharmacol..

[10] Shimizu K., Sasaki H., Hida H., Fujita H., Obinata K., Shikida M., Nagamori E. (2010). Assembly of skeletal muscle cells on a si-mems device and their generative force measurement. Biomed. Microdevices.

[11] Shimizu K., Fujita H., Nagamori E. (2013). Evaluation systems of generated forces of skeletal muscle cell-based bio-actuators. J. Biosci. Bioeng..

[12] Shimizu K., Araki H., Sakata K., Tonomura W., Hashida M., Konishi S. (2015). Microfluidic devices for construction of contractile skeletal muscle microtissues. J. Biosci. Bioeng..

[13] Vandenburgh H., Shansky J., Benesch-Lee F., Barbata V., Reid J., Thorrez L., Valentini R., Crawford G. (2008). Drug-screening platform based on the contractility of tissue-engineered muscle. Muscle Nerve.

[14] Sakar M.S., Neal D., Boudou T., Borochin M.A., Li Y.Q., Weiss R., Kamm R.D., Chen C.S., Asada H.H. (2012). Formation and optogenetic control of engineered 3D skeletal muscle bioactuators. Lab Chip.

[15] Fujita H., Shimizu K., Nagamori E. (2009). Novel method for fabrication of skeletal muscle construct from the C2C12 myoblast cell line using serum-free medium AIM-V. Biotechnol. Bioeng..

[16] Fujita H., Shimizu K., Nagamori E. (2010). Novel method for measuring active tension generation by C2C12 myotube using UV-crosslinked collagen film. Biotechnol. Bioeng..

[17] Madden L., Juhas M., Kraus W.E., Truskey G.A., Bursac N. (2014). Bioengineered human myobundles mimic clinical responses of skeletal muscle to drugs. eLIFE.

[18] Dennis R.G., Kosnik P.E. (2000). Excitability and isometric contractile properties of mammalian skeletal muscle constructs engineered in vitro. In Vitro Cell. Dev. Biol. Anim..

[19] Yamamoto Y., Ito A., Fujita H., Nagamori E., Kawabe Y., Kamihira M. (2011). Functional evaluation of artificial skeletal muscle tissue constructs fabricated by a magnetic force-based tissue engineering technique. Tissue Eng. Part A.

[20] McAleer C.W., Smith A.S.T., Najjar S., Pirozzi K., Long C.J., Hickman J.J. (2014). Mechanistic investigation of adult myotube response to exercise and drug treatment in vitro using a multiplexed functional assay system. J. Appl. Physiol..

[21] Sun Y., Duffy R., Lee A., Feinberg A.W. (2013). Optimizing the structure and contractility of engineered skeletal muscle thin films. Acta Biomater..

[22] Vandenburgh H., Shansky J., Benesch-Lee F., Skelly K., Spinazzola J.M., Saponjian Y., Tseng B.S. (2009). Automated drug screening with contractile muscle tissue engineered from dystrophic myoblasts. FASEB J..

[23] Massaccesi L., Goi G., Tringali C., Barassi A., Venerando B., Papini N. (2016). Dexamethasone-induced skeletal muscle atrophy increases o-glcnacylation in C2C12 cells. J. Cell. Biochem.

[24] Castillero E., Alamdari N., Lecker S.H., Hasselgren P.O. (2013). Suppression of atrogin-1 and murf1 prevents dexamethasone-induced atrophy of cultured myotubes. Metabolism.

[25] Bian W., Liau B., Badie N., Bursac N. (2009). Mesoscopic hydrogel molding to control the 3D geometry of bioartificial muscle tissues. Nat. Protoc..

[26] Latres E., Amini A.R., Amini A.A., Griffiths J., Martin F.J., Wei Y., Lin H.C., Yancopoulos G.D., Glass D.J. (2005). Insulin-like growth factor-1 (IGF-1) inversely regulates atrophy-induced genes via the phosphatidylinositol 3-kinase/akt/mammalian target of rapamycin (PI3K/Akt/mTOR) pathway. J. Biol. Chem..

[27] Fujita H., Hirano M., Shimizu K., Nagamori E. (2010). Rapid decrease in active tension generated by C2C12 myotubes after termination of artificial exercise. J. Muscle Res. Cell Motil..

[28] Fujita H., Nedachi T., Kanzaki M. (2007). Accelerated de novo sarcomere assembly by electric pulse stimulation in C2C12 myotubes. Exp. Cell. Res..

[29] Ikeda K., Ito A., Sato M., Kawabe Y., Kamihira M. (2016). Improved contractile force generation of tissue-engineered skeletal muscle constructs by IGF-I and Bcl-2 gene transfer with electrical pulse stimulation. Regen. Ther..

[30] Ito A., Yamamoto Y., Sato M., Ikeda K., Yamamoto M., Fujita H., Nagamori E., Kawabe Y., Kamihira M. (2014). Induction of functional tissue-engineered skeletal muscle constructs by defined electrical stimulation. Sci. Rep..

[31] Cohick W.S., Clemmons D.R. (1993). The insulin-like growth-factors. Annu. Rev. Physiol..

[32] Sato M., Ito A., Kawabe Y., Nagamori E., Kamihira M. (2011). Enhanced contractile force generation by artificial skeletal muscle tissues using IGF-I gene-engineered myoblast cells. J. Biosci. Bioeng..

[33] Huang Y.C., Dennis R.G., Larkin L., Baar K. (2005). Rapid formation of functional muscle in vitro using fibrin gels. J. Appl. Physiol..

[34] Sacheck J.M., Ohtsuka A., McLary S.C., Goldberg A.L. (2004). IGF-I stimulates muscle growth by suppressing protein breakdown and expression of atrophy-related ubiquitin ligases, atrogin-1 and murf1. Am. J. Physiol. -Endocrinol. Metab..

[35] Rommel C., Bodine S.C., Clarke B.A., Rossman R., Nunez L., Stitt T.N., Yancopoulos G.D., Glass D.J. (2001). Mediation of IGF-1-induced skeletal myotube hypertrophy by PI(3)K/Akt/mTOR and PI(3)K/Akt/GSK3 pathways. Nat. Cell. Biol..

[36] Tanaka A., Woltjen K., Miyake K., Hotta A., Ikeya M., Yamamoto T., Nishino T., Shoji E., Sehara-Fujisawa A., Manabe Y. (2013). Efficient and reproducible myogenic differentiation from human iPS cells: Prospects for modeling miyoshi myopathy in vitro. PLoS ONE.

[37] Hosoyama T., McGivern J.V., Van Dyke J.M., Ebert A.D., Suzuki M. (2014). Derivation of myogenic progenitors directly from human pluripotent stem cells using a sphere-based culture. Stem Cells Transl. Med..

[38] Chal J., Al Tanoury Z., Hestin M., Gobert B., Aivio S., Hick A., Cherrier T., Nesmith A.P., Parker K.K., Pourquie O. (2016). Generation of human muscle fibers and satellite-like cells from human pluripotent stem cells in vitro. Nat. Protoc..

[39] Biedasek K., Andres J., Mai K., Adams S., Spuler S., Fielitz J., Spranger J. (2011). Skeletal muscle 11beta-HSD1 controls glucocorticoid-induced proteolysis and expression of E3 ubiquitin ligases atrogin-1 and murf-1. PLoS ONE.

